# Dynamics based clustering of globin family members

**DOI:** 10.1371/journal.pone.0208465

**Published:** 2018-12-04

**Authors:** Dror Tobi

**Affiliations:** 1 Department of Molecular Biology, Ariel University, Ariel, Israel; 2 Department of Computer Sciences, Ariel University, Ariel, Israel; University of Tennessee, UNITED STATES

## Abstract

A methodology to cluster proteins based on their dynamics’ similarity is presented. For each pair of proteins from a dataset, the structures are superimposed, and the Anisotropic Network Model modes of motions are calculated. The twelve slowest modes from each protein are matched using a local mode alignment algorithm based on the local sequence alignment algorithm of Smith–Waterman. The dynamical similarity distance matrix is calculated based on the top scoring matches of each pair and the proteins are clustered using a hierarchical clustering algorithm. The utility of this method is exemplified on a dataset of protein chains from the globin family and a dataset of tetrameric hemoglobins. The results demonstrate the effect of the quaternary structure of globin members on their intrinsic dynamics and show good ability to distinguish between different states of hemoglobin, revealing the dynamical relations between them.

## Introduction

Protein structures are dynamic rather than static, and it is protein dynamics that play a key role in executing their functions [1–9]. Understanding the relation between protein function and dynamics is fundamental for comprehending the protein structure–dynamics–function relationship. Such an understanding can stem from a comparison of the dynamics of related proteins or the same protein in different states. Comparison of sequences and structures is a common approach in the study of proteins. BLAST [10] is by far the most widely used software for sequence similarity detection, and structure based comparison and classification algorithms like CATH [11], SCOP [12], and DALI [13] provide a good overview of the entire protein structure universe. In recent years, several tools and techniques have been developed for comparison of protein dynamics and contributed to the development of the field of comparative dynamics [14–16].

Many of the tools and techniques for dynamical comparison rely on analysis of low-frequency normal modes from coarse-grained elastic network models (ENM) or principal components analysis of biomolecular structures and dynamic simulation. These studies have proven useful in unraveling the collective modes and, in particular, those at the low frequency end of the mode spectrum that underlie protein equilibrium dynamics [17]. Simple and computationally efficient normal mode models were developed to study protein dynamics [18, 19]. A detailed structure of a protein is not necessary to obtain the global dynamics, rather the shape of the molecule plays a predominant role in determining the eigenvectors [20]. Even low-resolution on-lattice models can provide insights into functionally important global dynamics [21]. Low-frequency normal modes from coarse-grained ENMs have been shown to match experimentally observed conformational changes [22, 23]. Recently, using Anton supercomputing technology [24, 25], a comparison was made between PCA modes obtained from micro- to milli-second full atomic MD simulations and modes obtained from the Anisotropic Network Model (ANM) [26, 27]. Close overlap was found between the principal modes of these two techniques, reinforcing normal mode analysis as a tool for exploring protein dynamics [28].

Clustering algorithms rely on similarity (or distance) scores to identify closely related proteins. Sequence clustering algorithms used percentage sequence identity of aligned proteins to measure sequence similarity. In order to reduce computational cost, short word filtering of non-relevant sequences may precede the alignment step such as in BLAST [10] or CD-HIT [29]. Structure clustering algorithms use structural similarity scores such as the Root Mean Squares positional Deviation (RMSD) or TM-score [30, 31]; for comprehensive review see [32]. For example, the DALI server optimizes a structural alignment; that is, a sequential set of one-to-one correspondences between C^α^ atoms [33]. Similarly, there is a need to develop dynamical similarity scores; such scores can enable us to classify proteins according to their dynamics. Previously, Zen et al. developed a method to align proteins based on their equilibrium dynamics inferred from ENM [34]. Munts et al. showed that dynamics similarity can be measured by comparing Dynamic Fingerprint Matrix [35]. We previously showed that the similarity of normal modes of motion can be measured using alignment algorithms [36, 37] based on the global sequence alignment algorithm of Needleman-Wunsch [38]. The optimal way of comparing complex molecular motions is, however, far from trivial and no efficient methods were developed so far for large scale dynamics-based clustering of proteins.

A new dynamics similarity score (DSS) based on local alignment of ANM modes of motion is presented here. The algorithm is based on the local sequence alignment algorithms of Smith-Waterman [39]. Its ability and usefulness to measure dynamics similarity is demonstrated on the globin family of proteins. Globins are globular proteins comprising 6–8 α-helices (labelled A–H), with members distributed across all three domains of life: bacteria, archaea and eukaryotes. Each globin polypeptide binds a single molecule of iron-protoporphyrin-IX (Heme B) that can bind diatomic gaseous ligands such as O_2_, CO, NO, and other small ligands [40]. In mammals, hemoglobin (Hb) acts as an O_2_ carrier to transport O_2_ from lungs to tissues, while myoglobin (Mb) is responsible for intracellular O_2_ storage in muscles and its transport from the plasma membrane to mitochondria. The heteromeric quaternary structure provides the hemoglobin a mechanism for cooperative oxygen-binding and allosteric regulatory control [41], so the protein switches between two forms: tense (T) low-affinity state and relaxed (R) high-affinity state upon ligand binding [42]. There are two classical models proposed for describing the allosteric mechanism of Hb: the Monod, Wyman, and Changeux (MWC) concerted model [43] and the Koshland, Nemethy, and Filmer (KNF) sequential model [44]. After solving the R conformation the R2 conformation was discovered, this conformation more accurately represents the liganded Hb end state [45]. Two different structural sub-classes of the globin fold are recognized. The 3-on-3 fold is the canonical Hb fold, exemplified by Mb. The ‘3-on-3’ designation refers to the α-helical ‘sandwich’ formed by the A-G-H and B-E-F helices [46]. Members of this class include, for example: Hb, Mb, non-symbiotic Hb and protoglobin. The second structural class is the truncated Hb (trHb) class, also called ‘2-on-2’ Hbs, based on the arrangement of the B–E and G–H helical pairs [47].

The three-dimensional (3-D) structure of globins is well preserved, but their sequences are very different [48]. However, it was possible to identify, from a set of aligned protein structures, a core set of residues that are located at relatively invariant 3-D positions [49]. Maguid et al. [50] showed that two slowest Gaussian network model (GNM) normal modes of motions are conserved within this family, indicating common dynamics within the globin family. The present study demonstrates that the DSS can be used to cluster proteins based on their dynamics similarity. The DSS score matrix is calculated based on the top scoring matches of each pair of globin members and the proteins are clustered using a hierarchical clustering algorithm. The utility of this method is exemplified on a dataset for protein chains from the globin family and a dataset of tetrameric hemoglobins. The results demonstrate the effect of the quaternary structure of globin members on their intrinsic dynamics and the dynamical relations between different available Hb structures.

## Materials and methods

### Globin datasets

A list of protein structures was compiled based on the globins Superfamily (1.10.490.10) of the CATH database [11, 51]. Protein structures were downloaded from the Protein Data Bank (PDB) [52] and a total of 1030 structures and 1289 unique chains were obtained. Two datasets were created; the first (dataset1) contains 117 randomly selected globin chains. The second dataset (dataset2) consists of all 320 tetrameric Hb excluding three structures 1ITH, 4HRR and 4HRT that have a unique quaternary structure and do not superimpose with other Hbs. The PDB codes of two datasets are listed in S1 and S2 Tables (Supporting Information).

### ANM calculation

The ANM modes of motion of the superimposed structures were calculated as previously reported [27, 53, 54]. Each residue was represented by a single node positioned at its C^α^ atom and a cutoff distance of 15Å was used. Heme groups were represented as four nodes corresponding to CHA, CHB, CHC and CHD atoms. ANM modes were calculated to the biological unit. In the case when only a single chain was compared, and the chain was a part of the biological unit, only the part of the modes that corresponds to this chain was used in the alignment. Every pair of compared structures was superposed using the TM-align software [30, 31] prior to ANM calculations. Calculating ANM modes of motion to a large dataset, especially when some of the structures are large, is very time consuming. Therefore, fast ANM calculations of only the slowest 40 modes were performed using the Spectra library for large scale eigenvalue problems [55]. This library is a C++ implementation of ARPACK [56] and uses the Lanczos algorithm [57].

### Anisotropic network model modes local alignment

The commonly used Smith–Waterman [39] local sequence alignment algorithm was modified to align ANM modes with few modifications. ANM mode analysis results in a set of vectors $\left\{\vec{U}\right\}$ describing the deformation of residues from their equilibrium position (native structure) in the Cartesian space. Let $\vec{U_{i}^{k}}$ be the deformation vector of residue *i* in mode *k* of one protein and $\vec{V_{j}^{l}}$ the deformation vector of residue *j* in mode *l* of another protein. The score for residues *i* and *j* upon alignment of modes *k* and *l* is defined as:

$$S_{ij}=\frac{{\vec{U}}_{i}^{k}∙{\vec{V}}_{j}^{l}}{\left|{\vec{U}}_{i}^{k}||{\vec{V}}_{j}^{l}\right|}-C\;where\;0\leq C\leq 1$$ (1)

*S*_*ij*_ will be positive if their cosine value is greater than C that is the two deformation vectors pointing in the same direction and negative if their cosine is smaller than C. Here we used C = 0.7 radians (~40°) to define the threshold for vector similarity. In case of alignment of homologous or identical proteins, it is possible to guide the algorithm to prefer the matching of spatially close residues by applying distance constraints. Distance constraints were applied in the present work by modifying the alignment score *S*_*ij*_ as follows:

$$S_{ij}=\left\{ \begin{array}{l} \frac{{\vec{U}}_{i}^{k}∙{\vec{V}}_{j}^{l}}{\left|{\vec{U}}_{i}^{k}||{\vec{V}}_{j}^{l}\right|}-C,\;r_{ij}\leq R_{c}\\ -1\;,r_{ij}>R_{c} \end{array}\right.$$ (2)

where *r*_*ij*_ is the C^α^ distance between residues *i* and *j* and *R*_*c*_ is the cutoff distance set here to 10Å. Since the sign of the fluctuations in each mode is arbitrary, the alignment of two modes, *a* and *b*, is done twice. Once between the two original modes (*a* and *b*) and once between mode *a* and the negative of the second mode–*b*, with the best alignment (highest total score) being used.

For each pair from the *n* slowest aligned modes, an alignment matrix is created and the best non-overlapping (up to 200) gapless matches with minimal length of seven residues (gapless alignment of a single mode pair) are kept. The top *2n* matches (best scores) are selected and best *S*_*ij*_ is kept, for residues *i* and *j* of the first and second aligned proteins, for each mode combination. The final residue dynamical similarity score of each residue is the sum of all its best (kept) *S*_*ij*_. The sum of the average residue dynamical similarity score of both proteins divided by two is defined as their DSS. The ability of the current algorithm to identify local dynamics similarity is demonstrated in a recent paper [58] where we show a detailed dynamical comparison between myoglobin and hemoglobin.

### Clustering and principal component analysis

Clustering calculations were performed in R environment for statistical computing and graphics [59]. Clustering was performed using the hierarchical clustering function hclust with default parameters after converting the DSS matrix into a distance matrix. Preliminary calculation showed that both hclust and agnes functions give similar results. Principal Component Analysis was performed using the R package Bio3D [60] in combination with the MUSCLE program for multiple sequence alignment [61].

## Results

**Fig 1:**
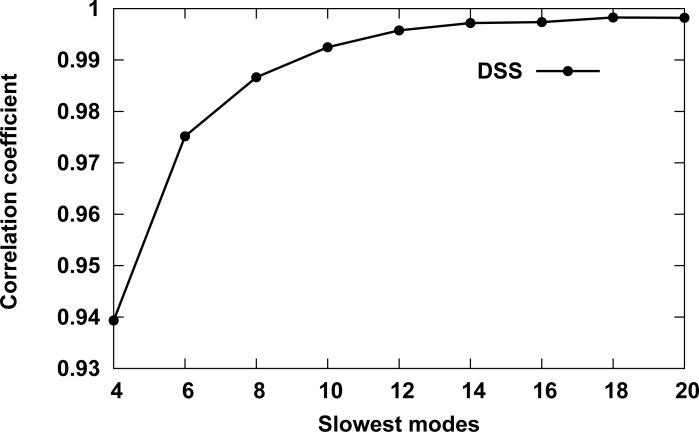
Correlation coefficient between DSSs calculated with different number of slow modes. The calculations were performed for globin chains in dataset1. The first data point (Slowest modes value 4) marks the correlation coefficients between DSS scores calculated using the 2 and 4 slowest modes, with the second between DSS scores calculated using the 4 and 6 slowest modes, and so on.

In order to determine the optimal number of slow modes to use for comparing the dynamics of globin family members, the DSSs between all chains in dataset1 were calculated using a series of slow nodes (*n* = 2,4,6, … 20). The correlation coefficient was calculated between successive DSSs and the results are depicted in Fig 1. The first data point marks the correlation coefficients between DSSs, calculated using the 2 and 4 slowest modes. The second between DSSs calculated using the 4 and 6 slowest modes, and so on. The more slow modes being used, the higher the correlation coefficient until it reaches a plateau around the 12 slowest modes. Therefore, the 12 slowest modes were used in the following calculations. The results also indicate that the algorithm is not very sensitive to the exact number of top *2n* matches (see Methods) that are used to calculate the DSSs.

### Clustering chains of globin members

**Fig 2:**
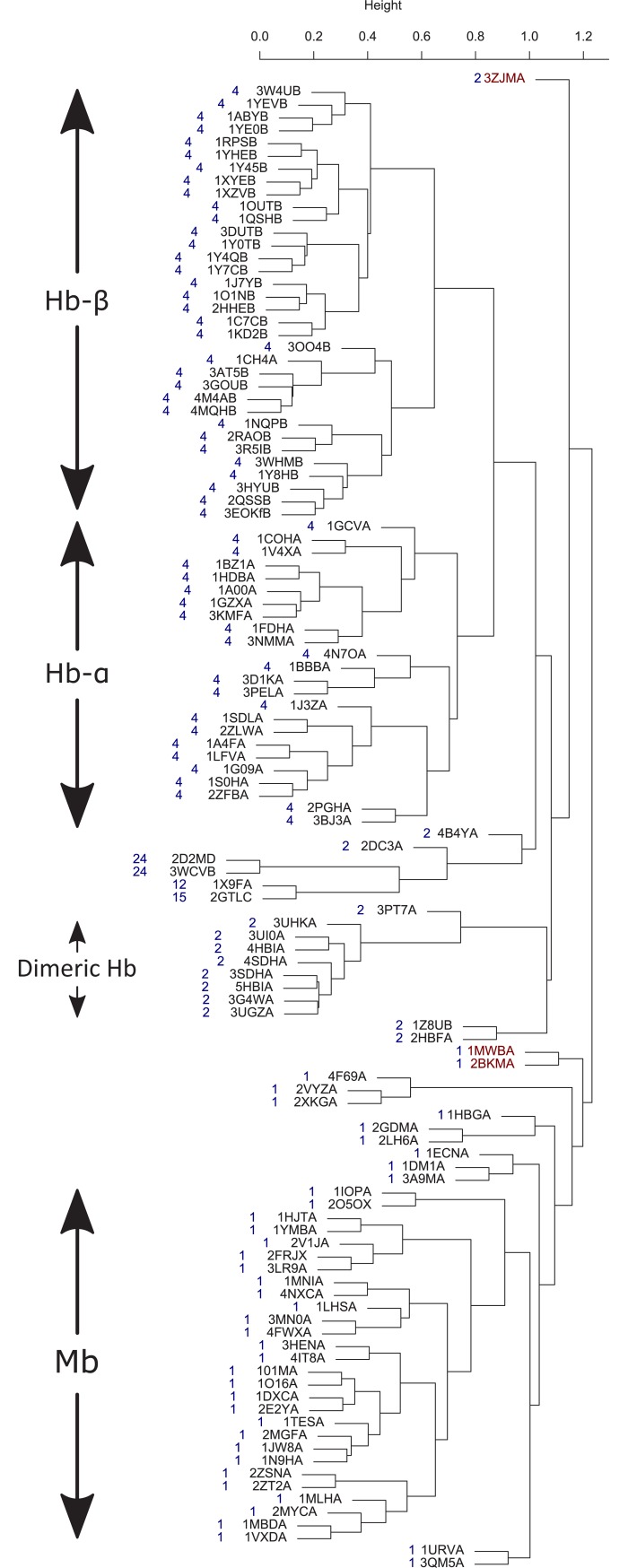
Globin chains (dataset1) dynamics similarity dendrogram. The number of chains in each biological assembly are indicated in blue next to each structure. Three outliers are highlighted in maroon.

Clustering of dataset1 was performed by using the DSS scores matrix and the dendrogram is depicted in Fig 2, with the number of chains in each biological assembly indicated in blue next to each structure. Overall, the dendrogram classification follows the number of chains in the biological assembly, indicating the strong effect of the quaternary structure on the dynamics of its subunits. The globin chains are divided into three major groups: Hb-α chains, Hb-β chains, and Mb and another small group of dimeric Hbs. There are three noticeable outliers (highlighted in maroon). The PDB code 3ZJM [62] (Fig 2 top) is dimeric protoglobin from *Methanosarcina acetivorans*, a strictly anaerobic methanogenic *Archaea*, whose biological role is still unknown. Protoglobin is the first globin identified in *Archaea* [63]. Another two noticeable outliers are the PDB codes 1MWB [64] and 2BKM [65] (Fig 2 middle); both these structures belong to the truncated hemoglobin family.

**Fig 3:**
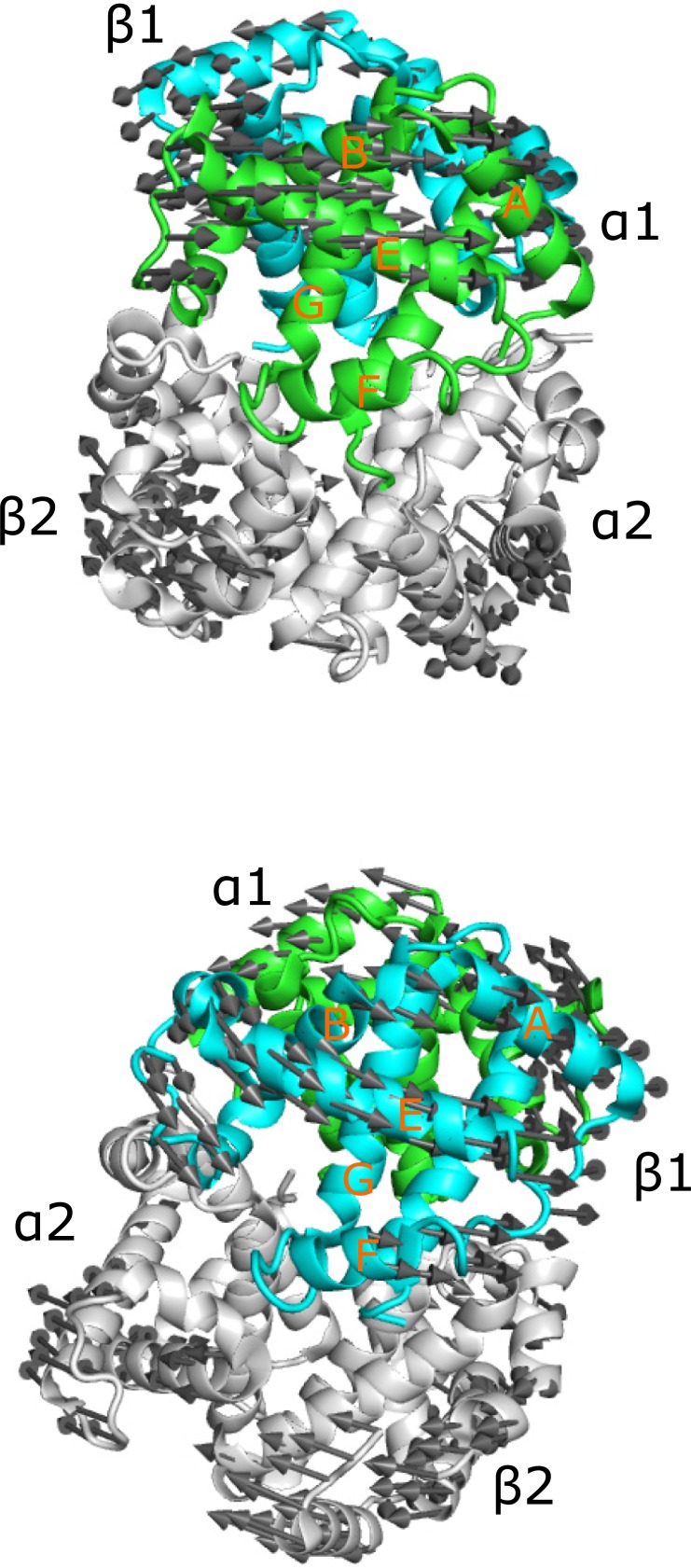
Porcupine plot of the first mode of motions of liganded Hb 1BBB[67]. The mode is depicted using the viewpoint of the α (top) and β (bottom) chains. The chains are colored green (α1), cyan (β1), white (α2, β2).

The fact that Hb α and β chains are clustered in different groups indicates that there is a difference in the dynamics of the two chains. The first mode of motion of Hb describes the R2 to T transition [66]. Fig 3 depicts this motion for the liganded human Hb 1BBB [67] using a porcupine plot from the viewpoint of the α (top) and β (bottom) chains. There are seven helices in α subunit of Hb and eight in β subunit, with the difference between them in the region that connects helices B and E in the α subunits. This region is composed of one short helix and a large loop in α subunit and a short helix-loop-short helix in β subunit; therefore, helix D is missing in the α chain. The 1BBB is presented using α- and β- subunits viewpoint with similar orientation of the helix E. The circular motion of α subunit is in approximately 30° off the helix E direction while the circular motion of β chain is parallel to helix E direction. That is, the two chains have distinguishable motion of the slowest mode and the difference increases with the second and third modes.

### Clustering tetrameric hemoglobins

The vast majority of the Hb proteins are tetrameric. These globin members were clustered separately in order to obtain a better understanding of their dynamical relations. A set of 320 tetrameric Hb structures were compiled (dataset2) from the CATH globin family and they are listed in S2 Table. PDB entries 1A3N (T) [68] and 1BBB (R2) [67] were used to represent human Hb in the unbound and bound states, respectively. The intact protein structures were superimposed prior to the ANM mode calculations, then the DSS scores matrix was calculated, and the proteins were clustered as before. The complete dendrogram is presented in S1 Fig. The resulting dendrogram divides the Hbs into two main groups, one includes the T-state and the other includes the R2-state structure. The nearest neighbors of the T and R2 state, they are highlighted in blue and maroon, respectively, and are listed in Table 1. For the majority of the proteins listed in the table, we were able to verify from the literature that they are indeed T- and R2 state structures. Two structures are distinguished as outliers in the dendrogram 3AT6 [69] and 2M6Z [70]. 3AT6 is the yellow-spotted river turtles (*Podocnemis unifilis*, *Pleurodira*, *Chelonia*, *REPTILIA*) adult Hb-A and the first refined model for reptilian adult Hb A structure. 2M6Z is an NMR solution structure of HbCO with overall quaternary structure more similar to the X-ray R structure of HbCO A than to the R2 structure. The authors concluded that it is a dynamic intermediate between the R and R2 forms. The dynamical comparison shows that this structure has unique dynamics distinct from the T and R2 states and is slightly closer to the T rather than R2 state (R state is referred below).

**Table 1:** DSS based nearest neighbors of hemoglobin T- and R2- state structures.

T state	R2 state
1Y4V	3B75
3HXN	1G0A
1XZ7	1SI4
1XY0	1M9P
1Y0C	1QXD
1Y35	1BBB
1Y85	3IC0
1DKE	1QXE
1A3N	3R5I
2D60	

**Fig 4:**
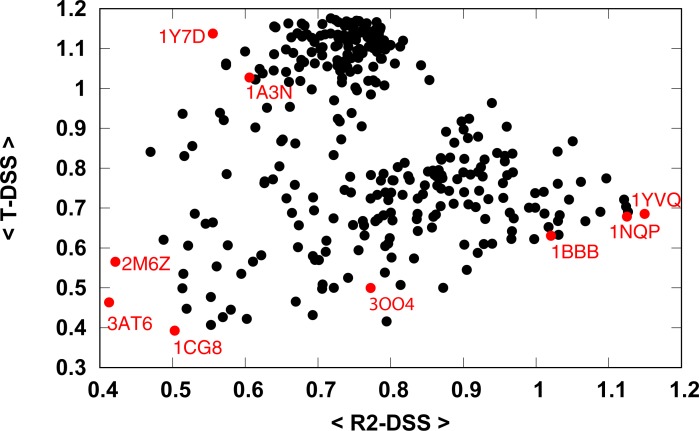
Hemoglobin dynamics similarity map to the T- and R2 states. The abscissa and the ordinate represent the average dynamics similarity score from the R2 and T states.

Dynamics based 2-D mapping of the tetrameric Hbs was carried out by calculating their average DSS from the T and R2 representatives (Table 1), with the results depicted in Fig 4. The R2 structure 1BBB has a high average R2-DSS and a low T-DSS, as expected. The two proteins that show the highest average R2-DSS are Hb-E structures 1YVQ and 1NQP [71]. The two structures are in the bound state, the former binds to CO and the latter to CN and was also reported to represent the R2 state. As expected, these proteins also have a low average T-DSS. The T structure 1A3N has a high average T-DSS and low average R2-DSS. The structure 1Y7D [72] has the highest average T-DSS and lowest average R2-DSS. This structure represents transitions referred to as T-to-T_High_ transitions between the quaternary-T structure of wild-type deoxyhemoglobin and an ensemble of related T-like quaternary structures that are induced by some mutations in the Trp37β cluster. The R-state structure 3OO4 [73] is located between the R2 and T state with average R2-DSS greater than the average T-DSS. The map shows a continuous path from the R2 to R to T states. The two outliers 3AT6 and 2M6Z show low average T- and R2- DSS, another such structure is 1CG8 [74]. 1CG8 is an Hb structure from *Dasyatis akajei*, a stingray that is one of the most distantly related vertebrate Hbs to human HbA. Larger structural deviations between *Dasyatis akajei* Hb and human HbA are observed in various parts of the molecule, even in the E and F helices. The average T- and R2- DSSs for all Hbs in dataset2 is provided in the S3 Table.

**Fig 5:**
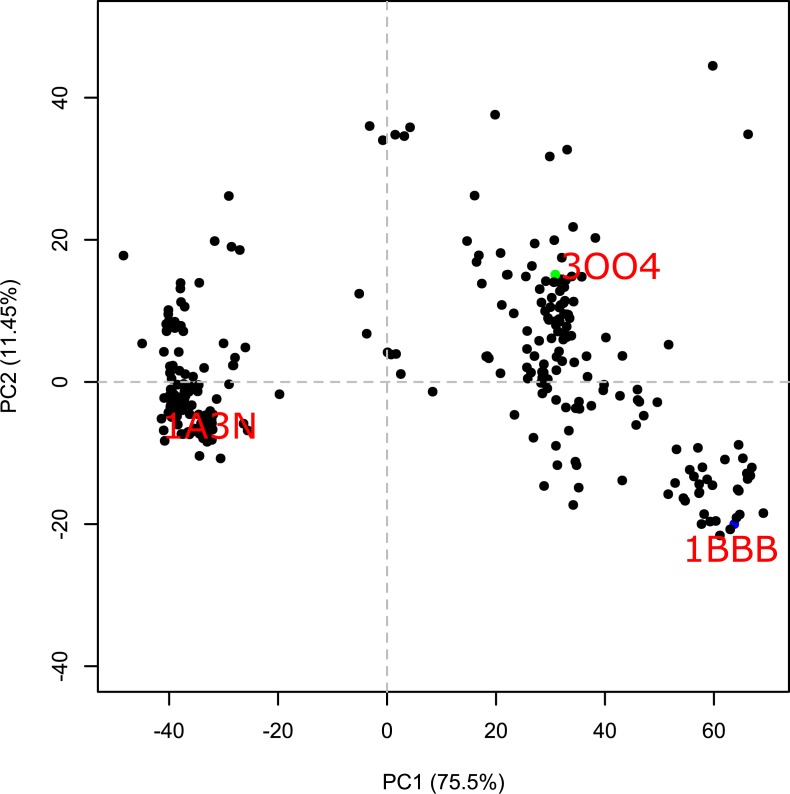
Hemoglobin principal component analysis. The abscissa and the ordinate represent the two main principal components with their variance written in parenthesis.

PCA analysis is a common method for 2-D mapping of the structural space. The resulting principal components (orthogonal eigenvectors) describe the axes of maximal variance of the distribution of structures. The 2D map of the tetrameric Hbs along the first two principal components is presented in Fig 5. Similarly, to the dynamics based 2-D mapping, the R2-state (1BBB) and T state are on opposite sides of the map and the R state is located closer to the R2 state than the T state. The main difference between the two maps is the clear gap between the cluster of structures that are at the vicinity of the T state and the rest of the structures. While, the dynamic based 2-D map shows intermediate states linking the two states.

### Dynamics similarity versus structural similarity

**Fig 6:**
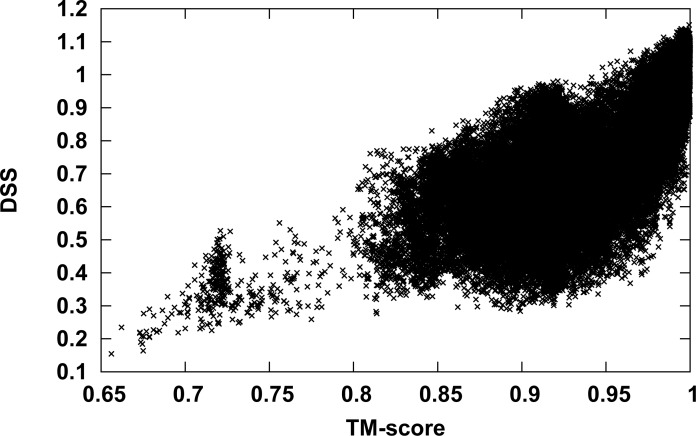
DSS as a function of the TM-score. The TM-scores (abscissa) versus the DSSs (ordinate) for all hemoglobin structures in dataset2 depicted using point plot.

ANM normal modes are derived from the protein structure. Hence, there is an expected relation between DSSs and structural similarity scores such as the TM-score obtained here during structural superposing of the Hb pairs. Fig 6 presents the DSSs as a function of the TM-score. The figure shows that Hb pairs with high DSS also have a high TM-score. However, the opposite is not always true: Protein pairs with high TM-score may have high or low DSS scores. One example is the 1CG8 structure; many Hb pairs, which include this structure, have a high TM-score and low DSS. Although the global 1CG8 structure is very close to other Hb structures, significant mutations and/or conformational changes are observed between this structure and HbA around the hemes, in the C-terminal region of the β-subunit, in the α1β2 interface, and in the organic phosphate-binding site of HbA [74]. These changes affect the DSS more than the TM-score.

Another way to compare between the DSS and the TM-score is a comparison of the derived dendrograms, the TM-score based dendrogram of dataset2 is presented in S2 Fig. Similarly, to the DSS-based dendrogram, the Hbs are divided into two major groups, one includes the T-state and the other include the R2-state. Branching of the major groups is different. The nearest neighbors of the T and R2 state according to the TM-score based dendrogram are listed in Table 2. The overlap with the DSS based nearest neighbors (Table 1) of the R2-state is higher than the T-state as the non-overlapping structures 1NQP and 1YVQ are in a close branch of the DSS-based dendrogram. Thus, the different scores resulting in differences in the dendrograms.

**Table 2:** TM-score based nearest neighbors of hemoglobin T- and R2- state structures.

T state	R2 state
3WCP	**1SI4**
1A3O	**3B75**
**1A3N**	**1BBB**
**2D60**	1NQP
2HHE	1YVQ
2D5Z	
4L7Y	

Overlapping DSS- and TM-score nearest neighbors are marked in bold.

## Discussion and conclusion

A novel methodology for dynamics-based clustering of proteins is presented here. The method performs local ANM mode alignments of *n* slowest modes, using an algorithm based on the local sequence alignment algorithms of Smith-Waterman [39]. Dynamical comparison of the globin members is challenging since the proteins are in different multimeric states ranging from monomers to 24-mers. Hence, some of the modes were inter-subunits and others intra-subunit motions. Despite this difficulty, we were able to develop a fully automated procedure that identifies the best matches between the slowest *n* modes of each protein without the need to select reference modes. Recently, Ponzoni et al. classified the LeuT-fold superfamily of secondary active transporters using the three slowest ANM modes calculated for the monomers/protomers [75]. This methodology necessitates the choice of a reference state and is harder to implement when the protomer in its multimeric state has significantly different dynamics.

Clustering of globin chains shows that Mb, Hb-α and Hb-β are clustered in different groups, indicating the unique dynamics of each group within the globin family. The dynamical difference between Mb and Hb subunits is expected as there is a functional difference between the two proteins; Mb has a higher affinity for oxygen than Hb and lacks allosteric and cooperative function. The distinguishable dynamics between α and β subunits of Hb indicate that the two subunits have an asymmetric role in agreement with the following studies. PELE molecular dynamics simulations of Hb showed that the ligand exit paths for the β-subunit are considerably different from those for the α-subunit [76]. Experimentally, geminate ligand recombination reaction studies showed dissimilar behavior of these two subunits, pointing to their nonequivalent role [77–79].

The clustering results of the globin chains reflect the quaternary structure of the chains. However, even clustering of tetrameric Hb, i.e. globins with the same quaternary structure, results in a meaningful dendrogram and a 2D dynamic similarity map of the different structures of Hb. The gap in Fig 4. of the dynamical based 2-D map between the T and R2 states suggests that there is an indirect path from the R2 to T states through the R, with the R state closer to R2. The map reflects the ability of the DSS to distinguish between the different Hb structures and correctly classify them. In the case of a compact molecular structure such as that of a globular protein, the shape of the molecule plays a predominant role in determining the eigenvectors of low-frequency normal modes [20, 21]. Therefore, the use of only one or two slow modes for calculating DSS can affect the ability of the algorithm to distinguish between similar structures. Based on the correlation analysis of DSSs calculated using different number of slow modes (Fig 1), the number *n* = 12 of slowest modes was chosen as the optimal one. This number is in agreement with the algorithm of Zen et al. [34] for dynamics-based alignment of proteins, which uses the 10 slowest modes.

Although elastic network model normal modes of motion are derived from protein structures, dynamics-based clustering is different from structure-based clustering. This is due to the fact that the modes of motions are calculated for the entire biological assembly even when we compare only a single chain. Therefore, we can take into account the effect of the quaternary structure on the intrinsic dynamics of the examined chain. In addition, as shown in Fig 6, proteins with high DSS have a high TM-score; however, proteins with a high TM-score may have high or low DSS. The present methodology has few limitations, first, it can only be used with structurally similarity proteins, since structural superimposing is necessary prior to ANM mode calculation. Second, normal mode analysis assumes a harmonic potential, but not all protein motions can be correctly described by harmonic potentials [80]. Thereby, the dynamics here only describe protein behaviors near the energy minimum. Proteins with moderate structural conservation can be compared by increasing *R*_*c*_. Dynamics comparison of structurally dissimilar proteins can be performed by comparing GNM modes since these modes do not depend on the specific pose of a structure in cartesian space [36, 81]. The information embedded in the GNM modes is more limited than ANM modes, hence, comparison of GNM vs. ANM modes can be viewed as low vs. high resolution dynamical comparisons [58]. Combination of the two methods will be necessary to achieve a broader applicability of this approach. As proteins depend on their dynamics to execute their function, dynamics-based alignment and clustering algorithms are expected to deepen the understanding of the structure-dynamics-function relation of proteins.

## Supporting information

S1 Table Dataset1. 117 randomly selected globin chains. The first 4 letters of each protein correspond to the PDB code of the structure and the fifth letter to the chain name.
(DOCX)

S2 Table Dataset2. 320 tetrameric hemoglobin PDB codes.
(DOCX)

S3 Table Tetrameric hemoglobins (dataset2) average T- and R2- DSSs. (XLSX)

S1 Fig Tetrameric hemoglobins (dataset2) dynamics similarity dendrogram. (PDF)

S2 Fig Tetrameric hemoglobins (dataset2) structural similarity dendrogram. (PDF)

## References

[1] Henzler-Wildman KA, Lei M, Thai V, Kerns SJ, Karplus M, Kern D. A hierarchy of timescales in protein dynamics is linked to enzyme catalysis. Nature. 2007;450(7171):913–6. 10.1038/nature06407 . 18026087 10.1038/nature06407

[2] Henzler-Wildman K, Kern D. Dynamic personalities of proteins. Nature. 2007;450(7172):964–72. Epub 2007/12/14. nature06522 [pii] 10.1038/nature06522 . 18075575 10.1038/nature06522

[3] Tobi D, Bahar I. Structural changes involved in protein binding correlate with intrinsic motions of proteins in the unbound state. Proc Natl Acad Sci U S A. 2005;102(52):18908–13. Epub 2005/12/16. 0507603102 [pii] 10.1073/pnas.0507603102 ; PubMed Central PMCID: PMC1323175. 16354836 10.1073/pnas.0507603102 PMC1323175

[4] Meireles L, Gur M, Bakan A, Bahar I. Pre-existing soft modes of motion uniquely defined by native contact topology facilitate ligand binding to proteins. Protein Sci. 2011 20(10):1645–58. Epub 2011/08/10. 10.1002/pro.711 . 21826755 10.1002/pro.711 PMC3218357

[5] Balog E, Perahia D, Smith JC, Merzel F. Vibrational softening of a protein on ligand binding. J Phys Chem B. 2011 115(21):6811–7. Epub 2011/05/11. 10.1021/jp108493g . 21553905 10.1021/jp108493g

[6] Zhuravlev PI, Papoian GA. Protein functional landscapes, dynamics, allostery: a tortuous path towards a universal theoretical framework. Q Rev Biophys. 2010;43(3):295–332. Epub 2010/09/08. S0033583510000119 [pii] 10.1017/S0033583510000119 . 20819242 10.1017/S0033583510000119

[7] Zheng W, Brooks BR, Thirumalai D. Allosteric transitions in biological nanomachines are described by robust normal modes of elastic networks. Curr Protein Pept Sci. 2009;10(2):128–32. Epub 2009/04/10. . 19355980 10.2174/138920309787847608 PMC3610319

[8] Kleinekathofer U, Isralewitz B, Dittrich M, Schulten K. Domain motion of individual F1-ATPase beta-subunits during unbiased molecular dynamics simulations. J Phys Chem A. 2011;115(25):7267–74. Epub 2011/04/02. 10.1021/jp2005088 ; PubMed Central PMCID: PMC3121902. 21452901 10.1021/jp2005088 PMC3121902

[9] Manglik A, Kobilka B. The role of protein dynamics in GPCR function: insights from the beta2AR and rhodopsin. Curr Opin Cell Biol. 2014;27:136–43. 10.1016/j.ceb.2014.01.008 ; PubMed Central PMCID: PMCPMC3986065. 24534489 10.1016/j.ceb.2014.01.008 PMC3986065

[10] Altschul SF, Gish W, Miller W, Myers EW, Lipman DJ. Basic local alignment search tool. J Mol Biol. 1990;215(3):403–10. Epub 1990/10/05. 10.1016/S0022-2836(05)80360-2 [pii]. . 2231712 10.1016/S0022-2836(05)80360-2

[11] Pearl FM, Bennett CF, Bray JE, Harrison AP, Martin N, Shepherd A, et al The CATH database: an extended protein family resource for structural and functional genomics. Nucleic Acids Res. 2003;31(1):452–5. Epub 2003/01/10. ; PubMed Central PMCID: PMC165509. 12520050 10.1093/nar/gkg062 PMC165509

[12] Andreeva A, Howorth D, Brenner SE, Hubbard TJ, Chothia C, Murzin AG. SCOP database in 2004: refinements integrate structure and sequence family data. Nucleic Acids Res. 2004;32(Database issue):D226–9. Epub 2003/12/19. 10.1093/nar/gkh039 [pii]. ; PubMed Central PMCID: PMC308773. 14681400 10.1093/nar/gkh039 PMC308773

[13] Holm L, Rosenstrom P. Dali server: conservation mapping in 3D. Nucleic Acids Res. 2010;38(Web Server issue):W545–9. Epub 2010/05/12. gkq366 [pii] 10.1093/nar/gkq366 ; PubMed Central PMCID: PMC2896194. 20457744 10.1093/nar/gkq366 PMC2896194

[14] Micheletti C. Comparing proteins by their internal dynamics: exploring structure-function relationships beyond static structural alignments. Phys Life Rev. 2013;10(1):1–26. Epub 2012/12/04. S1571-0645(12)00132-7 [pii] 10.1016/j.plrev.2012.10.009 . 23199577 10.1016/j.plrev.2012.10.009

[15] Fuglebakk E, Tiwari SP, Reuter N. Comparing the intrinsic dynamics of multiple protein structures using elastic network models. Biochim Biophys Acta. 2015;1850(5):911–22. 10.1016/j.bbagen.2014.09.021 . 25267310 10.1016/j.bbagen.2014.09.021

[16] Tiwari SP, Reuter N. Conservation of intrinsic dynamics in proteins-what have computational models taught us? Curr Opin Struct Biol. 2017;50:75–81. 10.1016/j.sbi.2017.12.001 . 29287233 10.1016/j.sbi.2017.12.001

[17] Kitao A, Go N. Investigating protein dynamics in collective coordinate space. Curr Opin Struct Biol. 1999;9(2):164–9. Epub 1999/05/14. S0959-440X(99)80023-2 [pii] 10.1016/S0959-440X(99)80023-2 . 10322205 10.1016/S0959-440X(99)80023-2

[18] Bahar I, Atilgan AR, Erman B. Direct evaluation of thermal fluctuations in proteins using a single-parameter harmonic potential. Fold Des. 1997;2(3):173–81. Epub 1997/01/01. 10.1016/S1359-0278(97)00024-2 . 9218955 10.1016/S1359-0278(97)00024-2

[19] Hinsen K. Analysis of domain motions by approximate normal mode calculations. Proteins. 1998;33(3):417–29. Epub 1998/11/26. 10.1002/(SICI)1097-0134(19981115)33:3<417::AID-PROT10>3.0.CO;2–8 [pii]. . 9829700 10.1002/(sici)1097-0134(19981115)33:3<417::aid-prot10>3.0.co;2-8

[20] Lu M, Ma J. The role of shape in determining molecular motions. Biophys J. 2005;89(4):2395–401. 10.1529/biophysj.105.065904 ; PubMed Central PMCID: PMCPMC1366739. 16055547 10.1529/biophysj.105.065904 PMC1366739

[21] Doruker P, Jernigan RL. Functional motions can be extracted from on-lattice construction of protein structures. Proteins. 2003;53(2):174–81. 10.1002/prot.10486 . 14517969 10.1002/prot.10486

[22] Hinsen K, Thomas A, Field MJ. Analysis of domain motions in large proteins. Proteins. 1999;34(3):369–82. Epub 1999/02/19. 10.1002/(SICI)1097-0134(19990215)34:3<369::AID-PROT9>3.0.CO;2-F [pii]. . 10024023

[23] Krebs WG, Alexandrov V, Wilson CA, Echols N, Yu H, Gerstein M. Normal mode analysis of macromolecular motions in a database framework: developing mode concentration as a useful classifying statistic. Proteins. 2002;48(4):682–95. Epub 2002/09/05. 10.1002/prot.10168 . 12211036 10.1002/prot.10168

[24] Klepeis JL, Lindorff-Larsen K, Dror RO, Shaw DE. Long-timescale molecular dynamics simulations of protein structure and function. Curr Opin Struct Biol. 2009;19(2):120–7. Epub 2009/04/14. S0959-440X(09)00037-2 [pii] 10.1016/j.sbi.2009.03.004 . 19361980 10.1016/j.sbi.2009.03.004

[25] Shaw DE, Maragakis P, Lindorff-Larsen K, Piana S, Dror RO, Eastwood MP, et al Atomic-level characterization of the structural dynamics of proteins. Science. 2010;330(6002):341–6. Epub 2010/10/16. 330/6002/341 [pii] 10.1126/science.1187409 . 20947758 10.1126/science.1187409

[26] Eyal E, Chennubhotla C, Yang LW, Bahar I. Anisotropic fluctuations of amino acids in protein structures: insights from X-ray crystallography and elastic network models. Bioinformatics. 2007;23(13):i175–84. Epub 2007/07/25. 23/13/i175 [pii] 10.1093/bioinformatics/btm186 . 17646294 10.1093/bioinformatics/btm186

[27] Atilgan AR, Durell SR, Jernigan RL, Demirel MC, Keskin O, Bahar I. Anisotropy of fluctuation dynamics of proteins with an elastic network model. Biophys J. 2001;80(1):505–15. Epub 2001/02/13. S0006-3495(01)76033-X [pii] 10.1016/S0006-3495(01)76033-X ; PubMed Central PMCID: PMC1301252. 11159421 10.1016/S0006-3495(01)76033-X PMC1301252

[28] Gur M, Zomot E, Bahar I. Global motions exhibited by proteins in micro- to milliseconds simulations concur with anisotropic network model predictions. J Chem Phys. 2013;139(12):121912 Epub 2013/10/05. 10.1063/1.4816375 ; PubMed Central PMCID: PMC3739829. 24089724 10.1063/1.4816375 PMC3739829

[29] Huang Y, Niu B, Gao Y, Fu L, Li W. CD-HIT Suite: a web server for clustering and comparing biological sequences. Bioinformatics. 2010;26(5):680–2. 10.1093/bioinformatics/btq003 ; PubMed Central PMCID: PMCPMC2828112. 20053844 10.1093/bioinformatics/btq003 PMC2828112

[30] Zhang Y, Skolnick J. Scoring function for automated assessment of protein structure template quality. Proteins. 2004;57(4):702–10. Epub 2004/10/12. 10.1002/prot.20264 . 15476259 10.1002/prot.20264

[31] Zhang Y, Skolnick J. TM-align: a protein structure alignment algorithm based on the TM-score. Nucleic Acids Res. 2005;33(7):2302–9. Epub 2005/04/26. 33/7/2302 [pii] 10.1093/nar/gki524 ; PubMed Central PMCID: PMC1084323. 15849316 10.1093/nar/gki524 PMC1084323

[32] Hasegawa H, Holm L. Advances and pitfalls of protein structural alignment. Curr Opin Struct Biol. 2009;19(3):341–8. 10.1016/j.sbi.2009.04.003 . 19481444 10.1016/j.sbi.2009.04.003

[33] Holm L, Laakso LM. Dali server update. Nucleic Acids Res. 2016;44(W1):W351–5. 10.1093/nar/gkw357 ; PubMed Central PMCID: PMCPMC4987910. 27131377 10.1093/nar/gkw357 PMC4987910

[34] Zen A, Carnevale V, Lesk AM, Micheletti C. Correspondences between low-energy modes in enzymes: dynamics-based alignment of enzymatic functional families. Protein Sci. 2008;17(5):918–29. Epub 2008/03/29. ps.073390208 [pii] 10.1110/ps.073390208 ; PubMed Central PMCID: PMC2327282. 18369194 10.1110/ps.073390208 PMC2327282

[35] Munz M, Lyngso R, Hein J, Biggin PC. Dynamics based alignment of proteins: an alternative approach to quantify dynamic similarity. BMC Bioinformatics. 2010;11:188 Epub 2010/04/20. 1471-2105-11-188 [pii] 10.1186/1471-2105-11-188 ; PubMed Central PMCID: PMC2868010. 20398246 10.1186/1471-2105-11-188 PMC2868010

[36] Tobi D. Dynamics alignment: comparison of protein dynamics in the SCOP database. Proteins. 2012;80(4):1167–76. Epub 2012/01/26. 10.1002/prot.24017 . 22275069 10.1002/prot.24017

[37] Tobi D. Dynamical differences of hemoglobin and the ionotropic glutamate receptor in different states revealed by a new dynamics alignment method. Proteins. 2017;85(8):1507–17. 10.1002/prot.25311 . 28459140 10.1002/prot.25311

[38] Needleman SB, Wunsch CD. A general method applicable to the search for similarities in the amino acid sequence of two proteins. J Mol Biol. 1970;48(3):443–53. Epub 1970/03/01. 0022-2836(70)90057-4 [pii]. . 5420325 10.1016/0022-2836(70)90057-4

[39] Smith TF, Waterman MS. Identification of common molecular subsequences. J Mol Biol. 1981;147(1):195–7. . 7265238 10.1016/0022-2836(81)90087-5

[40] Gell DA. Structure and function of haemoglobins. Blood Cells Mol Dis. 2018;70:13–42. 10.1016/j.bcmd.2017.10.006 . 29126700 10.1016/j.bcmd.2017.10.006

[41] Storz JF, Opazo JC, Hoffmann FG. Gene duplication, genome duplication, and the functional diversification of vertebrate globins. Mol Phylogenet Evol. 2013;66(2):469–78. 10.1016/j.ympev.2012.07.013 ; PubMed Central PMCID: PMCPMC4306229. 22846683 10.1016/j.ympev.2012.07.013 PMC4306229

[42] Lukin JA, Kontaxis G, Simplaceanu V, Yuan Y, Bax A, Ho C. Quaternary structure of hemoglobin in solution. Proc Natl Acad Sci U S A. 2003;100(2):517–20. 10.1073/pnas.232715799 ; PubMed Central PMCID: PMCPMC141027. 12525687 10.1073/pnas.232715799 PMC141027

[43] Monod J, Wyman J, Changeux JP. On the Nature of Allosteric Transitions: A Plausible Model. J Mol Biol. 1965;12:88–118. Epub 1965/05/01. . 14343300 10.1016/s0022-2836(65)80285-6

[44] Koshland DE Jr., Nemethy G, Filmer D. Comparison of experimental binding data and theoretical models in proteins containing subunits. Biochemistry. 1966;5(1):365–85. Epub 1966/01/01. PMID: 5938952. 5938952 10.1021/bi00865a047

[45] Schumacher MA, Zheleznova EE, Poundstone KS, Kluger R, Jones RT, Brennan RG. Allosteric intermediates indicate R2 is the liganded hemoglobin end state. Proc Natl Acad Sci U S A. 1997;94(15):7841–4. ; PubMed Central PMCID: PMCPMC21516. 9223274 10.1073/pnas.94.15.7841 PMC21516

[46] Holm L, Sander C. Structural alignment of globins, phycocyanins and colicin A. FEBS Lett. 1993;315(3):301–6. . 8422921 10.1016/0014-5793(93)81183-z

[47] Pesce A, Couture M, Dewilde S, Guertin M, Yamauchi K, Ascenzi P, et al A novel two-over-two alpha-helical sandwich fold is characteristic of the truncated hemoglobin family. EMBO J. 2000;19(11):2424–34. 10.1093/emboj/19.11.2424 ; PubMed Central PMCID: PMCPMC212751. 10835341 10.1093/emboj/19.11.2424 PMC212751

[48] Lesk AM, Chothia C. How different amino acid sequences determine similar protein structures: the structure and evolutionary dynamics of the globins. J Mol Biol. 1980;136(3):225–70. . 7373651 10.1016/0022-2836(80)90373-3

[49] Altman RB, Gerstein M. Finding an average core structure: application to the globins. Proc Int Conf Intell Syst Mol Biol. 1994;2:19–27. . 7584390

[50] Maguid S, Fernandez-Alberti S, Ferrelli L, Echave J. Exploring the common dynamics of homologous proteins. Application to the globin family. Biophys J. 2005;89(1):3–13. Epub 2005/03/08. S0006-3495(05)72652-7 [pii] 10.1529/biophysj.104.053041 ; PubMed Central PMCID: PMC1366528. 15749782 10.1529/biophysj.104.053041 PMC1366528

[51] Dawson NL, Lewis TE, Das S, Lees JG, Lee D, Ashford P, et al CATH: an expanded resource to predict protein function through structure and sequence. Nucleic Acids Res. 2017;45(D1):D289–D95. 10.1093/nar/gkw1098 ; PubMed Central PMCID: PMCPMC5210570. 27899584 10.1093/nar/gkw1098 PMC5210570

[52] Berman H, Henrick K, Nakamura H. Announcing the worldwide Protein Data Bank. Nature structural biology. 2003;10(12):980 10.1038/nsb1203-980 . 14634627 10.1038/nsb1203-980

[53] Doruker P, Atilgan AR, Bahar I. Dynamics of proteins predicted by molecular dynamics simulations and analytical approaches: application to alpha-amylase inhibitor. Proteins. 2000;40(3):512–24. Epub 2000/06/22. 10.1002/1097-0134(20000815)40:3<512::AID-PROT180>3.0.CO;2-M [pii]. . 10861943

[54] Hensen U, Meyer T, Haas J, Rex R, Vriend G, Grubmuller H. Exploring protein dynamics space: the dynasome as the missing link between protein structure and function. PLoS One. 2012;7(5):e33931 Epub 2012/05/19. 10.1371/journal.pone.0033931 PONE-D-11-18707 [pii]. ; PubMed Central PMCID: PMC3350514. 22606222 10.1371/journal.pone.0033931 PMC3350514

[55] Qiu Y. Spectra. Available from: https://spectralib.org/.

[56] ARPACK SOFTWARE. Available from: http://www.caam.rice.edu/software/ARPACK/.

[57] Lanczos C. An iteration method for the solution of the eigenvalue problem of linear differential and integral operators. J Res Nat’l Bur 1950;45:255–82. Epub 282.

[58] Aharoni R, Tobi D. Dynamical comparison between Myoglobin and Hemoglobin. Proteins. 2018 10.1002/prot.25598 . 30183107 10.1002/prot.25598

[59] The R Project for Statistical Computing. Available from: https://www.r-project.org/.

[60] Grant BJ, Rodrigues AP, ElSawy KM, McCammon JA, Caves LS. Bio3d: an R package for the comparative analysis of protein structures. Bioinformatics. 2006;22(21):2695–6. 10.1093/bioinformatics/btl461 . 16940322 10.1093/bioinformatics/btl461

[61] Edgar RC. MUSCLE: multiple sequence alignment with high accuracy and high throughput. Nucleic Acids Res. 2004;32(5):1792–7. 10.1093/nar/gkh340 ; PubMed Central PMCID: PMCPMC390337. 15034147 10.1093/nar/gkh340 PMC390337

[62] Pesce A, Tilleman L, Donne J, Aste E, Ascenzi P, Ciaccio C, et al Structure and haem-distal site plasticity in Methanosarcina acetivorans protoglobin. PLoS One. 2013;8(6):e66144 10.1371/journal.pone.0066144 ; PubMed Central PMCID: PMCPMC3680402. 23776624 10.1371/journal.pone.0066144 PMC3680402

[63] Pesce A, Bolognesi M, Nardini M. Protoglobin: structure and ligand-binding properties. Adv Microb Physiol. 2013;63:79–96. 10.1016/B978-0-12-407693-8.00003-0 . 24054795 10.1016/B978-0-12-407693-8.00003-0

[64] Falzone CJ, Christie Vu B, Scott NL, Lecomte JT. The solution structure of the recombinant hemoglobin from the cyanobacterium Synechocystis sp. PCC 6803 in its hemichrome state. J Mol Biol. 2002;324(5):1015–29. . 12470956 10.1016/s0022-2836(02)01093-8

[65] Ilari A, Kjelgaard P, von Wachenfeldt C, Catacchio B, Chiancone E, Boffi A. Crystal structure and ligand binding properties of the truncated hemoglobin from Geobacillus stearothermophilus. Arch Biochem Biophys. 2007;457(1):85–94. 10.1016/j.abb.2006.09.033 . 17126283 10.1016/j.abb.2006.09.033

[66] Xu C, Tobi D, Bahar I. Allosteric changes in protein structure computed by a simple mechanical model: hemoglobin T<—>R2 transition. J Mol Biol. 2003;333(1):153–68. Epub 2003/10/01. S002228360301060X [pii]. . 14516750 10.1016/j.jmb.2003.08.027

[67] Silva MM, Rogers PH, Arnone A. A third quaternary structure of human hemoglobin A at 1.7-A resolution. J Biol Chem. 1992;267(24):17248–56. . 1512262

[68] Tame JR, Vallone B. The structures of deoxy human haemoglobin and the mutant Hb Tyralpha42His at 120 K. Acta Crystallogr D Biol Crystallogr. 2000;56(Pt 7):805–11. . 10930827 10.1107/s0907444900006387

[69] Hasegawa T, Shishikura F, Kuwada T. Side-necked turtle (Pleurodira, Chelonia, reptilia) hemoglobin: cDNA-derived primary structures and X-ray crystal structures of Hb A. IUBMB Life. 2011;63(3):188–96. 10.1002/iub.429 . 21445850 10.1002/iub.429

[70] Fan JS, Zheng Y, Choy WY, Simplaceanu V, Ho NT, Ho C, et al Solution structure and dynamics of human hemoglobin in the carbonmonoxy form. Biochemistry. 2013;52(34):5809–20. 10.1021/bi4005683 ; PubMed Central PMCID: PMCPMC4013309. 23901897 10.1021/bi4005683 PMC4013309

[71] Dasgupta J, Sen U, Choudhury D, Datta P, Chakrabarti A, Chakrabarty SB, et al Crystallization and preliminary X-ray structural studies of hemoglobin A2 and hemoglobin E, isolated from the blood samples of beta-thalassemic patients. Biochem Biophys Res Commun. 2003;303(2):619–23. . 12659864 10.1016/s0006-291x(03)00379-6

[72] Kavanaugh JS, Rogers PH, Arnone A. Crystallographic evidence for a new ensemble of ligand-induced allosteric transitions in hemoglobin: the T-to-T(high) quaternary transitions. Biochemistry. 2005;44(16):6101–21. 10.1021/bi047813a . 15835899 10.1021/bi047813a

[73] Yi J, Thomas LM, Musayev FN, Safo MK, Richter-Addo GB. Crystallographic trapping of heme loss intermediates during the nitrite-induced degradation of human hemoglobin. Biochemistry. 2011;50(39):8323–32. 10.1021/bi2009322 ; PubMed Central PMCID: PMCPMC3209482. 21863786 10.1021/bi2009322 PMC3209482

[74] Chong KT, Miyazaki G, Morimoto H, Oda Y, Park SY. Structures of the deoxy and CO forms of haemoglobin from Dasyatis akajei, a cartilaginous fish. Acta Crystallogr D Biol Crystallogr. 1999;55(Pt 7):1291–300. . 10393295 10.1107/s0907444999005934

[75] Ponzoni L, Zhang S, Cheng MH, Bahar I. Shared dynamics of LeuT superfamily members and allosteric differentiation by structural irregularities and multimerization. Philos Trans R Soc Lond B Biol Sci. 2018;373(1749). 10.1098/rstb.2017.0177 ; PubMed Central PMCID: PMCPMC5941172. 29735731 10.1098/rstb.2017.0177 PMC5941172

[76] Lucas MF, Guallar V. An atomistic view on human hemoglobin carbon monoxide migration processes. Biophys J. 2012;102(4):887–96. 10.1016/j.bpj.2012.01.011 ; PubMed Central PMCID: PMCPMC3283813. 22385860 10.1016/j.bpj.2012.01.011 PMC3283813

[77] Esquerra RM, Goldbeck RA, Reaney SH, Batchelder AM, Wen Y, Lewis JW, et al Multiple geminate ligand recombinations in human hemoglobin. Biophys J. 2000;78(6):3227–39. 10.1016/S0006-3495(00)76859-7 ; PubMed Central PMCID: PMCPMC1300904. 10827999 10.1016/S0006-3495(00)76859-7 PMC1300904

[78] Lepeshkevich SV, Karpiuk J, Sazanovich IV, Dzhagarov BM. A kinetic description of dioxygen motion within alpha- and beta-subunits of human hemoglobin in the R-state: geminate and bimolecular stages of the oxygenation reaction. Biochemistry. 2004;43(6):1675–84. 10.1021/bi034928q . 14769045 10.1021/bi034928q

[79] Birukou I, Maillett DH, Birukova A, Olson JS. Modulating distal cavities in the alpha and beta subunits of human HbA reveals the primary ligand migration pathway. Biochemistry. 2011;50(34):7361–74. 10.1021/bi200923k ; PubMed Central PMCID: PMCPMC3160505. 21793487 10.1021/bi200923k PMC3160505

[80] Ramanathan A, Savol AJ, Langmead CJ, Agarwal PK, Chennubhotla CS. Discovering conformational sub-states relevant to protein function. PLoS One. 2011;6(1):e15827 10.1371/journal.pone.0015827 ; PubMed Central PMCID: PMCPMC3030567. 21297978 10.1371/journal.pone.0015827 PMC3030567

[81] Tobi D. Large-scale analysis of the dynamics of enzymes. Proteins. 2013;81:1910–8. Epub 2013/06/06. 10.1002/prot.24335 . 23737241 10.1002/prot.24335

