# Supplementary material for: Dynamics based clustering of globin family members
Source: PLoS One. 2018 Dec 4;13(12):e0208465. doi: 10.1371/journal.pone.0208465 (PMC6279032; doi:10.1371/journal.pone.0208465)
Supplement: S1 Table — 117 randomly selected globin chains. The first 4 letters of each protein correspond to the PDB code of the structure and the fifth letter to the chain name. (DOCX) [file pone.0208465.s001.docx]

| 101MA | 1J3ZA | 1URVA | 2FRJX | 3AT5B | 3UHKA |
| --- | --- | --- | --- | --- | --- |
| 1A00A | 1J7YB | 1V4XA | 2GDMA | 3BJ3A | 3UI0A |
| 1A4FA | 1JW8A | 1VXDA | 2GTLC | 3D1KA | 3W4UB |
| 1ABYB | 1KD2B | 1X9FA | 2HBFA | 3DUTB | 3WCVB |
| 1BBBA | 1LFVA | 1XYEB | 2HHEB | 3EOKB | 3WHMB |
| 1BZ1A | 1LHSA | 1XZVB | 2LH6A | 3G4WA | 3ZJMC |
| 1C7CB | 1MBDA | 1Y0TB | 2MGFA | 3GOUB | 4B4YA |
| 1CH4A | 1MLHA | 1Y45B | 2MYCA | 3HENA | 4F69A |
| 1COHA | 1MNIA | 1Y4QB | 2O5OX | 3HYUB | 4FWXA |
| 1DM1A | 1MWBA | 1Y7CB | 2PGHA | 3KMFA | 4HBIA |
| 1DXCA | 1N9HA | 1Y8HB | 2QSSB | 3LR9A | 4IT8A |
| 1ECNA | 1NQPB | 1YE0B | 2RAOB | 3MN0A | 4M4AB |
| 1FDHA | 1O16A | 1YEVB | 2V1JA | 3NMMA | 4MQHB |
| 1G09A | 1O1NB | 1YHEB | 2VYZA | 3OO4B | 4N7OA |
| 1GCVA | 1OUTB | 1YMBA | 2XKGA | 3PELA | 4NXCA |
| 1GZXA | 1QSHB | 1Z8UB | 2ZFBA | 3PT7A | 4SDHA |
| 1HBGA | 1RPSB | 2BKMA | 2ZLWA | 3QM5A | 5HBIA |
| 1HDBA | 1S0HA | 2D2MD | 2ZSNA | 3R5IB |  |
| 1HJTA | 1SDLA | 2DC3A | 2ZT2A | 3SDHA |  |
| 1IOPA | 1TESA | 2E2YA | 3A9MA | 3UGZA |  |

**S1 Table**: Dataset1 - 117 randomly selected globin chains. The first 4 letters of each protein correspond to the PDB code of the structure and the fifth letter to the chain name.
