# Supplementary material for: Dynamics based clustering of globin family members
Source: PLoS One. 2018 Dec 4;13(12):e0208465. doi: 10.1371/journal.pone.0208465 (PMC6279032; doi:10.1371/journal.pone.0208465)
Supplement: S2 Table — 320 tetrameric hemoglobin PDB codes. (DOCX) [file pone.0208465.s002.docx]

| 1A00 | 1G09 | 1J7Y | 1RQ3 | 1Y22 | 1YGD | 2RAO | 3GKV | 3R5I |
| --- | --- | --- | --- | --- | --- | --- | --- | --- |
| 1A01 | 1G0A | 1JEB | 1RQ4 | 1Y2Z | 1YGF | 2RI4 | 3GOU | 3S65 |
| 1A0U | 1G0B | 1JY7 | 1RQA | 1Y31 | 1YH9 | 2W6V | 3GQG | 3S66 |
| 1A0Z | 1G9V | 1K0Y | 1RVW | 1Y35 | 1YHE | 2W72 | 3GQP | 3VRE |
| 1A3N | 1GBU | 1K1K | 1S0H | 1Y45 | 1YHR | 2YRS | 3GQR | 3VRF |
| 1A3O | 1GBV | 1KD2 | 1S5X | 1Y46 | 1YIE | 2Z6N | 3GYS | 3VRG |
| 1A4F | 1GCV | 1LA6 | 1S5Y | 1Y4B | 1YIH | 2ZFB | 3HF4 | 3W4U |
| 1A9W | 1GCW | 1LFL | 1SDK | 1Y4F | 1YVQ | 2ZLT | 3HHB | 3WCP |
| 1AJ9 | 1GLI | 1LFQ | 1SDL | 1Y4G | 1YVT | 2ZLU | 3HRW | 3WHM |
| 1B86 | 1GZX | 1LFT | 1SHR | 1Y4P | 1YZI | 2ZLV | 3HXN | 3WTG |
| 1BAB | 1HAB | 1LFV | 1SI4 | 1Y4Q | 2AA1 | 2ZLW | 3HYU | 4ESA |
| 1BBB | 1HAC | 1LFY | 1SPG | 1Y4R | 2B7H | 2ZLX | 3IC0 | 4G51 |
| 1BIJ | 1HBA | 1LFZ | 1THB | 1Y4V | 2D5X | 3A0G | 3IC2 | 4H2L |
| 1BUW | 1HBB | 1LJW | 1UIW | 1Y5F | 2D5Z | 3AT5 | 3K8B | 4HHB |
| 1BZ0 | 1HBH | 1M9P | 1V4U | 1Y5J | 2D60 | 3AT6 | 3KMF | 4IRO |
| 1BZ1 | 1HBR | 1MKO | 1V4W | 1Y5K | 2DHB | 3B75 | 3LQD | 4L7Y |
| 1BZZ | 1HBS | 1NEJ | 1V4X | 1Y7C | 2DN1 | 3BCQ | 3MJP | 4M4A |
| 1C7B | 1HCO | 1NIH | 1V75 | 1Y7D | 2DN2 | 3BJ1 | 3MJU | 4M4B |
| 1CBL | 1HDA | 1NQP | 1VWT | 1Y7G | 2DN3 | 3BJ2 | 3MKB | 4MQC |
| 1CBM | 1HDB | 1NS6 | 1WMU | 1Y7Z | 2DXM | 3BJ3 | 3NFE | 4MQG |
| 1CG5 | 1HDS | 1NS9 | 1XQ5 | 1Y83 | 2H8D | 3BOM | 3NG6 | 4MQH |
| 1CG8 | 1HGA | 1O1I | 1XXT | 1Y85 | 2H8F | 3CIU | 3NL7 | 4MQI |
| 1CH4 | 1HGB | 1O1K | 1XY0 | 1Y8H | 2HBS | 3CY5 | 3NMM | 4MQJ |
| 1CLS | 1HGC | 1O1O | 1XYE | 1Y8I | 2HCO | 3D17 | 3ODQ | 4MQK |
| 1CMY | 1HHO | 1OUT | 1XZ2 | 1Y8K | 2HHB | 3D1A | 3ONZ | 4N7N |
| 1COH | 1HV4 | 1OUU | 1XZ4 | 1Y8W | 2HHD | 3D1K | 3OO4 | 4N7O |
| 1DKE | 1I3D | 1PBX | 1XZ5 | 1YDZ | 2HHE | 3D4X | 3OO5 | 4N7P |
| 1DXT | 1I3E | 1QI8 | 1XZ7 | 1YE0 | 2M6Z | 3D7O | 3P5Q | 4N8T |
| 1DXU | 1IBE | 1QPW | 1XZU | 1YE2 | 2MHBf | 3DHR | 3PEL | 4NI0 |
| 1DXV | 1IWH | 1QSH | 1XZV | 1YEN | 2PEGf | 3DHT | 3PI8 | 4NI1 |
| 1FAW | 1J3Y | 1QSI | 1Y09 | 1YEO | 2PGH | 3DUT | 3PI9 | 4ODC |
| 1FDH | 1J3Z | 1QXD | 1Y0A | 1YEQ | 2QMB | 3EOK | 3PIA | 6HBW |
| 1FHJ | 1J40 | 1QXE | 1Y0C | 1YEU | 2QSP | 3EU1 | 3QJB |  |
| 1FN3 | 1J41 | 1R1X | 1Y0D | 1YEV | 2QSS | 3FH9 | 3QJC |  |
| 1FSX | 1J7S | 1R1Y | 1Y0T | 1YFF | 2QU0 | 3FS4 | 3QJD |  |
| 1G08 | 1J7W | 1RPS | 1Y0W | 1YG5 | 2R1H | 3GDJ | 3QJE |  |

**S2 Table: Dataset2.** 320 tetrameric Hemoglobin PDB codes.
