# Supplementary figures and images for: Dynamics based clustering of globin family members
Source: PLoS One. 2018 Dec 4;13(12):e0208465. doi: 10.1371/journal.pone.0208465 (PMC6279032; doi:10.1371/journal.pone.0208465)

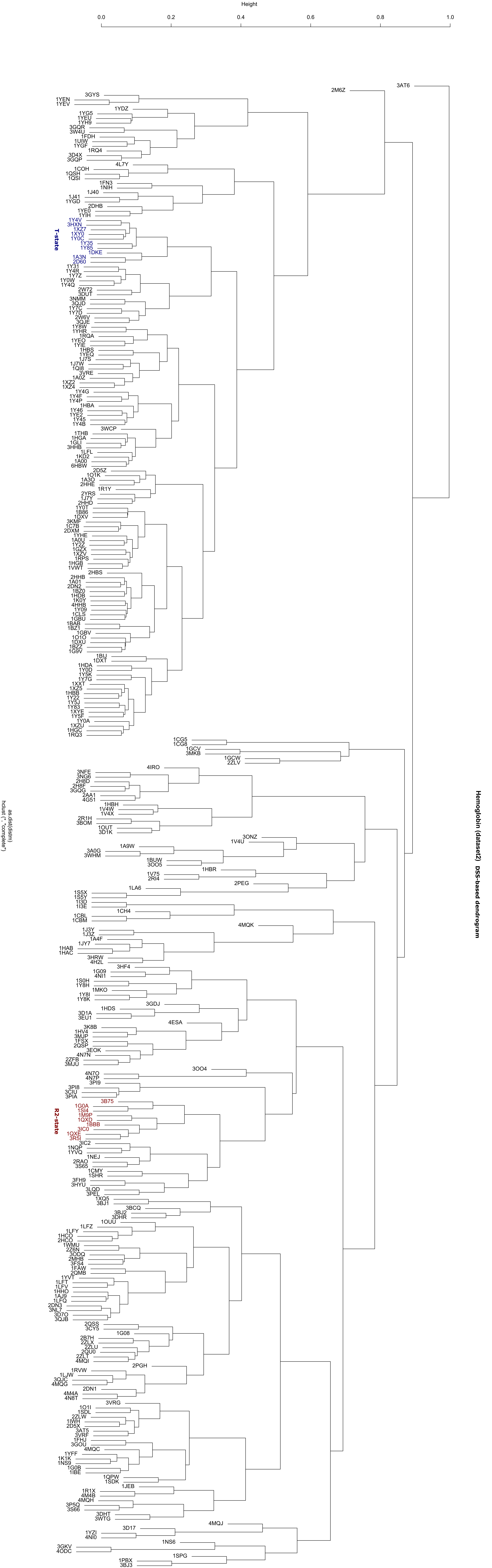

Supplement: S1 Fig — (PDF) [file pone.0208465.s004.pdf]

Height

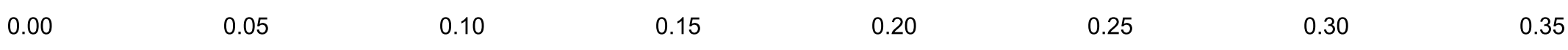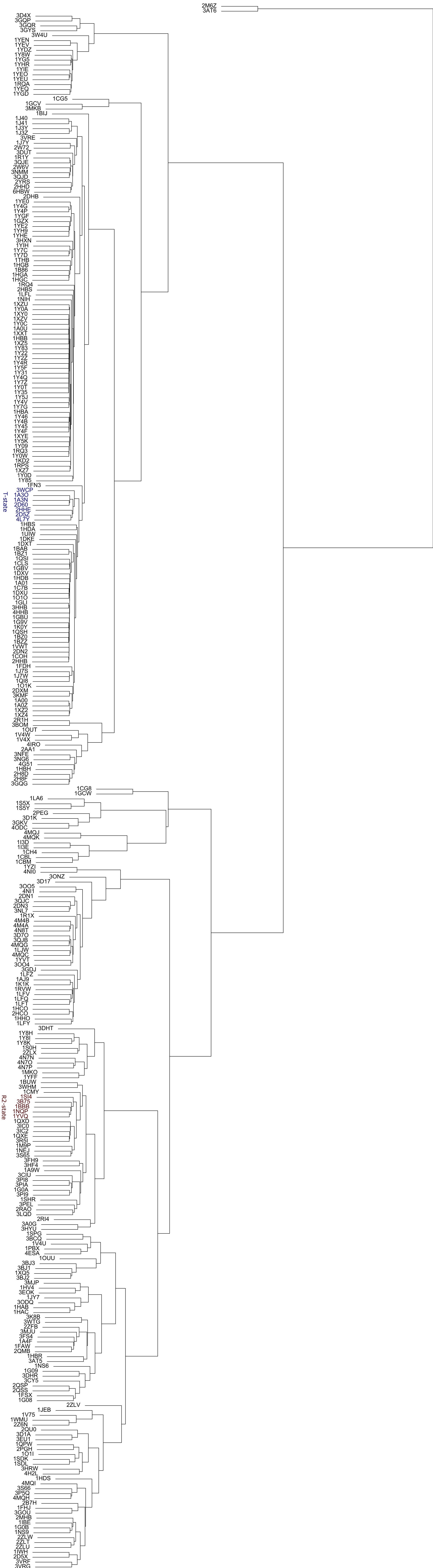

Hemoglobin (dataset2) TM-score based dendrogram

Supplement: S2 Fig — (PDF) [file pone.0208465.s005.pdf]
